# Biodiesel and activated carbon from arabica spent coffee grounds

**DOI:** 10.1016/j.mex.2023.102185

**Published:** 2023-04-14

**Authors:** Jefry Kusuma, Yuli S. Indartono, Didin Mujahidin

**Affiliations:** a Faculty of Mechanical and Aerospace Engineering, Bandung Institute of Technology, Bandung, Indonesia; b Faculty of Mathematics and Natural Science, Bandung Institute of Technology, Bandung, Indonesia

**Keywords:** Biodiesel, Activated carbon, Esterification, Transesterification, Carbonization, Adsorption, Transesterification for biodiesel and chemical activation for activated carbon

## Abstract

This study aims to analyze the potential and characteristics of biodiesel and activated carbon from spent coffee grounds (SCG).

• Biodiesel was obtained by extracting oil from SCG using Soxhlet extraction method with n-hexane solvent with an oil yield of 18.14% w/w of dry SCG. Furthermore, the coffee oil was esterified and transesterified to produce biodiesel with 57.32 % yield of coffee oil and higher heating value of 36.69 MJ/kg, density (15°C) of 0.89 g/mL, kinematic viscosity (40°C) of 7.67 mm^2^/s, acid number of 1.19 mg KOH/g oil.
• The residue in form of grounds after oil extraction process was turned into activated carbon using two step activation process. Carbonization process was carried out at 500, 600, 700, and 800°C for 30 minutes and then chemically activated using potassium hydroxide (KOH) at 750°C for 2 hours. As the comparison, activated carbon was also made from SCG without oil extraction process. This study shows that the adsorption capacity of activated carbon made from SCG with oil extraction was better than without oil extraction. The best adsorption of activated carbon was obtained from SCG with oil extraction and carbonized at 700°C with iodine value of 1,224.59 mg/g and methylene blue value of 153.08 mg/g.

## Graphical abstract

A graphical abstract is mandatory. The graphical abstract should summarize the contents of your article in a concise, pictorial form. Authors must provide images that clearly represent the work described in the article. Graphical abstracts should be submitted as a separate file. Image size: ***please provide an image with a minimum of 531 × 1328 pixels (h × w) or proportionally more***. The image should be readable at a size of 5 × 13 cm using a regular screen resolution of 96 dpi. Preferred file types: TIFF, EPS, PDF or MS Office files.

**Figure:**
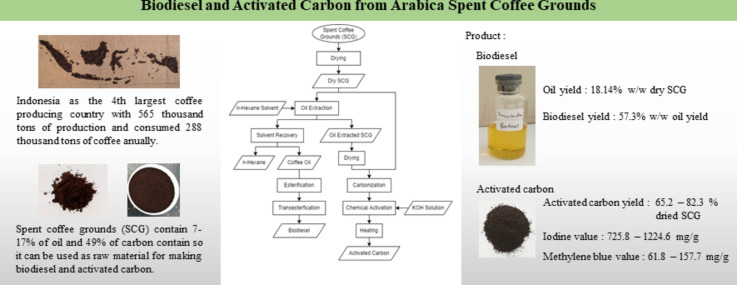

Specifications table

**Table:** 

Subject area:	Energy
More specific subject area:	Renewable energy
Name of your method:	Transesterification for biodiesel and chemical activation for activated carbon
Name and reference of original method:	N.A
Resource availability:	N.A

## Method details

### Introduction

Oil depletion is an ongoing problem and a way out must be sought by finding sources of fuel from renewable sources, such as biofuels in the form of biodiesel or bioethanol. Biodiesel can be produced from vegetable oil through squeezing or extraction process such as palm oil, coconut oil, and jatropha oil. On the other hand, bioethanol can be derived from materials containing sugar and starch such as sugarcane molasses, corn, palm sugar, sago, and cassava [1]. However, the United Nations (UN) noted an increase in the number of hunger figures from 2015. Until 2018 there were more than 821 million people in the world who suffer from hunger, food insecurity and malnutrition [2]. Seeing this problem, currently there is a second generation of biofuels, namely fuels made from non-food materials, waste or by-products from these foodstuffs, such as straw, bagasse, husks, palm empty bunches, and spent coffee grounds. Coffee was the second largest commodity traded in world trade after crude oil that makes the availability of spent coffee grounds (SCG) abundant. Indonesia as the 4^th^ largest coffee producing country in the world with total production of 565.08 thousand tons and consumption of 288 thousand tons in 2018/2019 makes the availability of spent coffee grounds abundant [3].

The amount of fat contained in coffee ranges from 7-17 % depending on the type of coffee, arabica coffee has oil content of 15% and 10% for robusta coffee [4]. In spent coffee grounds, the oil content ranged from 15 - 21.5% depending on the extraction method used [5]. This result is quite significant when compared to biodiesel raw materials in general, such as rapeseed oil (37 - 50%), palm oil (20%), and soybean oil (20%) [6]. Besides being used to produce biodiesel, spent coffee grounds also have ≥ 50% carbon content so they have good potential to be used as activated carbon [7].

Drying process plays a very important role in oil extraction. Decreasing the water content will increase the effectiveness of the solvent [8]. High water content will inhibit / interfere the penetration of the solvent and the oil diffusion process because the solvent used for oil extraction is not soluble in water [9]. However, very low water content will also decrease the amount of oil that can be extracted due to the reduced solubility of phosphatides in the absence of water [10,11]. The thickness of the sample to be dried also has a large effect on the drying rate. The time required for the drying process was directly proportional to the thickness of the sample and inversely proportional to the heating temperature [10,12].

Oil extraction process from spent coffee grounds can be done using various types of solvents, both polar solvents such as acetone, isopropanol, ethanol, methanol, and non-polar solvents such as n-pentane, hexane, toluene, n-octane, n-heptane, and chloroform [13,14]. Table 1 shows the oil yield using Soxhlet extraction method when hexane is used as the solvent from other studies.

**Table 1:** Oil yield from Soxhlet extraction using hexane as solvent reported in other studies.

Study	Dry spent coffee grounds to solvent ratio, m/v	Extraction time (h)	Oil yield on dry spent coffee grounds weight basis, m/m (%)
Abdullah and Bullent Koc [15]	1 : 15	8	13
Al-Hamamre et al. [14]	1 : 4.2	0.25 – 0.5	11.2 – 15.28
Ahangari and Sargolzaei [16]	1 : 15	6	16.7
Caetano et al. [13]	1 : 20	2.5 – 9.5	16
Couto et al. [17]	-	-	18.3
Deligiannis et al. [18]	-	-	10 - 15
Efthymiopoulos et al. [10]	1 : 4.44 – 1 : 8.88	1 - 2	12.55 – 15.69
Haile [19]	-	4-8	15.6
Kondamudi et al. [20]	1 : 3	1	13.4

Main composition of spent coffee grounds oil is palmitic acid and linoleic acid, followed by oleic acid and stearic acid. Some researchers reported a small composition of lauric acid, myristic acid, linolenic acid and arachidic acid. Table 2 shows the composition of spent coffee grounds oil and other plant oils obtained by previous researchers. The composition of the oil will affect the quality and properties of the oil produced.

**Table 2:** Composition of spent coffee grounds oil and other plant oils reported in other study.

	Lauric acid (C12:0)	Myristic acid (C14:0)	Palmitic acid (C16:0)	Stearic acid (C18:0)	Oleic acid (C18:1)	Linoleic acid (C18:2)	Linolenic acid (C18:3)	Arachidic acid (C20:0)	Others
Arabica spent coffee grounds oil									
Haile [19]	-	-	35.8%	8.1%	13.9%	37.3%	-	3.2%	1.7%
Haile et al. [21]	-	-	37.6%	7.6%	12,.7%	39.8%	-	–	2.3%
Oliveira et al. [22]	-	-	34%	7%	9%	1.5%	44%	3%	1%
Speer and Kölling-Speer [4]	-	-	26.6% - 27.8%	5.6% - 6.3%	6.7% - 8.2%	52.2% - 54.3%	2.2% - 2.6%	2.6% - 2.8%	-
Somnuk et al. [23]	0.02%	-	34.44%	-	7.74%	43.12%	-	2.83%	11.85%
Vardon et al. [24]			33.9%	7.3%	8.3%	45%	1.5%	2.5%	1%
Spent coffee grounds oil									
Ahangari and Sargolzaei [16]	3.54%	1.97%	43.61%	6.58%	8.15%	32.41%	1.3%	2.44%	-
Jin et al. [8]	-	-	35.7%	7.1%	9.4%	43.7%	-	2.2%	1.9%
Patra et al. [25]	-	-	35.7%	8.1%		37.3%	-		18.9%
Oil from other plants [26,27]									
Rapeseed oil	-	-	3.5%	0.9%	64.4%	22.3%	8.2%	-	0.7%
Olive oil	-	-	9.2%	3.4%	80.4%	4.5%	0.6%	-	1.9%
Sunflower oil	-	-	6.1%	3.3%	16.9%	73.7%	-	-	-
Soybean oil	-	0.1%	10.6%	4.8%	22.5%	52.3%	8.2%	-	1.5%
Palm oil	-	-	35%	-	7%	44%	14%	-	
Corn oil	-	-	11.6%	-	1.8%	25.1%	60.6%	0.4%	0.5%
Cottonseed oil	-	-	28.3%	-	0.8%	13.2%	57.5%	-	0.3%
Peanut oil	-	-	11.3%	-	2.3%	48.2%	31.9%	0.9%	5.4%

Oil obtained from the extraction process cannot be directly used as biodiesel, this oil needs to go through an esterification and transesterification process to convert it into FAME (Fatty Acid Methyl Ester). The final result of FAME is highly influenced by the quality and composition of the extracted oil. Oils with FFA level greater than 0.5% by weight need to go through a pretreatment stage to reduce FFA levels [28]. The esterification process can be used as a pretreatment stage which aims to convert free fatty acids into esters using alcohol and an acid catalyst [29]. FFA levels above 0.5% are reported to affect the product produced in the transesterification process. FFA levels above 5% will result in ester conversion below 90% [30]. Lowering FFA levels from 3.6% to 0.5% will increase the yield of biodiesel from 73% to 87% [31]. The ability of esterification and transesterification is strongly influenced by the type of alcohol used, the longer carbon chain of the alcohol used, the lower the esterification and transesterification performance. The transesterification performance obtained from various types of alcohol in sequence is methanol > ethanol > 2-propanol > 2-butanol > 2-hexanol > 2-octanol > 1-decanol. This is due to the difference in polarity, the polarity of the alcohol decreases as the number of carbon contained in the alcohol chain increases [32].

Esterification and transesterification processes require the presence of a catalyst. The acid catalysts that can be used in the esterification process are H_2_SO_4_, HCl, dan H_3_PO_4_ which are the most common acid homogeneous catalysts used in the esterification process. This catalyst is insensitive to FFA and H_2_O, but capable for catalyzing the esterification process of FFA. This homogeneous acid catalyst is corrosive to metallic materials and cannot be reused [33]. NaOH, KOH, and CH_3_ONa are homogeneous alkaline catalysts that are generally used in the biodiesel transesterification process because they have a fast reaction in short time [34]. However, this catalyst is sensitive to fatty acids and H_2_O in oil so that hydrolysis and saponification reactions can occur [35]. Spent coffee grounds biodiesel produced by previous researchers was summarized in Table 3.

**Table 3:** Properties of biodiesel from spent spent coffee grounds by previous researchers.

Study	Desity at 15°C (g/mL)	Kinematic viscosity at 40°C (mm^2^/s)	Acid number (mg KOH/g)	Iodine number (%-w (g-I_2_/100g))	Oxidation stability (h)	HHV (MJ/kg)
Caetano et al. [13]	0.911	12.88	2.14	46.5	-	-
Deligiannis et al. [18]	0.8943	5.612	0.36	-	7.9	39.49
Haile et al. [21]	0.8915	5.26	0.78	73.41	-	38.4
Haile [19]	0.88	5.4	0.7	74	-	39.6
Kondamudi et al. [20]	-	5.84	0.35	-	3.05	-
Oliveira et al. [36]	0.8941	4.9	-	-	-	38.414
Vardon et al. [24]	0.892	5.19	0.11	-	0.2	39.6

Activated carbon can be made from various kinds of raw materials, generally divided into two, namely from fossil raw materials (coal and petroleum residue), and from biomass-based carbon (wood, coconut shell, and agricultural residues) [37]. Spent coffee grounds was one of the lignocellulosic residues that are produced in large quantities throughout the world because coffee is the second largest trading commodity in the world after crude oil makes this waste easily found and widely available. There are two methods of carbon activation, one-step activation and two-step activation process.

## Material and methods

### Material

Spent coffee grounds used in this study are arabica spent coffee grounds from espresso machine obtained from Starbucks café located in Paris Van Java, Bandung, Indonesia. Potassium hydroxide, hydrochloric acid, potassium iodide, potassium iodate, sodium carbonate, sodium thiosulfate pentahydrate, starch soluble, and methylene blue were analytical grade from Merck. Phosphoric acid, n-hexane, sodium hydroxide, methanol, sulfuric acid, and aquadest were technical grade. Iodine used in this study was laboratory grade.

### Drying

The drying process was carried out by using 5 kg of spent coffee grounds which divided into 5 containers or trays, each tray contains 10 mm thickness of SCG and dried at 105 ± 5°C. Every 30 minutes a weight measurement was carried out for the five trays and rotation of the trays is done in order to obtain even drying results. The drying process was stopped after there was no decrease in mass in the five tray. The principle of this process was to calculate the mass loss on heating. To determine the moisture content contained in the spent coffee grounds before and after the drying process, it was validated using a volumetric Karl Fischer titrator.

### Oil production

The process of extracting oil from the dried spent coffee grounds was carried out using Soxhlet extractor and n-hexane as solvent, extraction was carried out in 5 cycles. This was done because after the 5^th^ cycle, the amount of oil produced was very small (≤ 0.03 g). From this process, a mixture of n-hexane and coffee oil will be collected and separated by using rotary evaporator to get coffee oil and n-hexane that can be reused for next extraction.

### Biodiesel production

The process of making biodiesel from coffee oil begins with the degumming process. The degumming process was carried out using phosphoric acid (H_3_PO_4_) then sodium hydroxide (NaOH) solvent and heated at 70°C for 1 hour. After the degumming process was complete, the mass, acid number, and saponification value of the oil were measured. If the oil acid number greater than 5 mg-KOH/g, it is necessary to do the esterification process because the levels of free fatty acids (FFA) are still high. The esterification process was carried out by mixing oil with methanol and sulfuric acid (H_2_SO_4_) as acid catalyst and heated for 1 hour at 60°C. The amount of methanol and sulfuric acid used based on formula below.

$$V_{methanol}=\frac{SV_{oil}\;}{Mr_{KOH}}\times \;m_{oil}\times 10\times \frac{Mr_{methanol}}{\rho_{methanol}}$$

$$V_{H_{2}SO_{4}}=1,5\%\;\times \;V_{oil}$$

with :

$V_{methanol}$ = volume of methanol used (mL)
$SV_{oil}$ = saponification value from oil (mg-KOH/g oil)
$Mr_{\mathrm{KOH}}$ = molar masss of KOH (g/mol)
$m_{\mathrm{oil}}$ = mass of oil (kg)
$Mr_{\mathrm{oil}}$ = molar mass of methanol (g/mol)
$\rho_{\mathrm{methanol}}$ = density of methanol (g/cm^3^)
$V_{H_{2}SO_{4}}$ = volume of sulfuric acid used (mL)
$V_{oil}$ = volume of oil (mL)

After the esterification process, the oil was washed with distilled water and the acid number of the oil was measured. If the acid number is > 5 mg-KOH/g, then the esterification process must be repeated. If the acid number is below 5 mg-KOH / g, then the mass and saponification value of oil were measured for further use in the transesterification process. The transesterification process was carried out by mixing the oil after esterification / oil with an acid number < 5 mg-KOH/g with methanol and potassium hydroxide (KOH) catalyst. This mixture was heated at 60°C for 1 hour and transesterified oil then washed with distilled water. The amount of methanol and potassium hydroxide used based on formula below.

$$V_{methanol}=\frac{SV_{oil}\;}{Mr_{KOH}}\times \;m_{oil}\times 2\times \frac{Mr_{methanol}}{\rho_{methanol}}$$

$$V_{\text{KOH}}=1,2\%\;\times \;V_{oil}$$

with :

$V_{methanol}$ = volume of methanol used (mL)
$SV_{oil}$ = saponification value from oil (mg-KOH/g oil)
$Mr_{\mathrm{KOH}}$ = molar masss of KOH (g/mol)
$m_{\mathrm{oil}}$ = mass of oil (kg)
$Mr_{\mathrm{methanol}}$ = molar masss of methanol (g/mol)
$\rho_{\mathrm{methanol}}$ = density of methanol (g/cm^3^)
$V_{KOH}$ = volume of potassium hydroxide used (mL)
$V_{oil}$ = volume oil (mL)

### Oil and biodiesel composition analysis

The types and levels of composition from the compounds contained in coffee oil was analyzed using Gas Chromatography and Mass Spectrometer (GC-MS). This study use Agilent 8890 with autosampler as gas chromatography instrument and Agilent 59778 as mass spectrometer instrument, Agilent HP-5MS column (capillary column : 30 m x 0.25 mm i.d. x 0.25 µm), and helium gas. Oven temperature 60°C, 15°C/m to 175°C, 2°C/m to 240°C, 10 minutes. Detector temperature of 230°C, and injector temperature of 250°C.

### Carbonization

Spent coffee grounds after the oil extraction and without oil extraction are processed into activated carbon to determine the effect of oil extraction on the quality of the activated carbon produced. The process of making activated carbon begins with the carbonization process carried out at temperature of 500, 600, 700, and 800°C at a rate of 10°C/min. after this temperature was attained, the reaction was held for 30 minutes and then it was cooled to ambient temperature. This process was carried out under inert condition by injecting nitrogen gas at 1 L/min.

### Activation process

Carbon from previous step (carbonization) was activated using KOH solution with concentration of 50%-mass as activating agent. The impregnation ratio was 1:4 m/m and impregnation was held for 2 hours. After impregnation, this mixture was dried at 105 ± 5°C for 6 hours and then heated in muffle furnace at 750°C at a rate of 10°C/min in inert condition. The heating process was held for 2 hours and then cooled to room temperature. Activated carbon obtained then washed using HCl 0.1M solution and rinsed with distilled water until neutral pH.

### Analysis of biodiesel and activated carbon properties

Some properties of biodiesel was measured using different standard, such as density at 15°C (ASTM D 1298), kinematic viscosity at 40°C (ASTM D 445), acid value (AOCS Cd 3d-63), saponification value (AOCS Ca 14-56), iodine value (AOCS Cd 1-25), oxidation stability (EN 14112), and higher heating value (ASTM D 240-02 (2007)). Properties from activated carbon was measured using ASTM standard, moisture content (ASTM D 2867 – 04), ash content (ASTM D 2866 – 94), volatile matter content (ASTM D 5832 – 98), and iodine number (ASTM D 4607 – 94).

## Results and discussion

### Drying

**Fig. 1:**
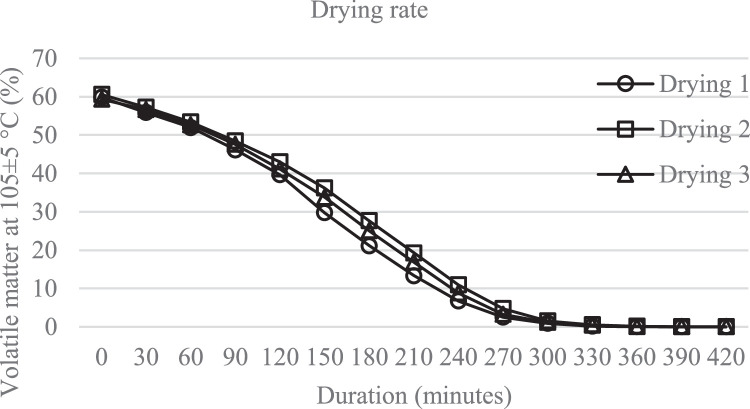
Drying rate.

The drying process was carried out 3 times with the same amount of spent coffee grounds. Fig. 1 shows the drying profile of spent coffee grounds.

By using Karl Fischer titrator, it was found that the moisture content of the spent coffee grounds before drying was 61.59% and after drying was 2.65%. This result was relevant because the water content was reduced by 58.94% compared to drying process above that shows the amount of substance that evaporates from drying was 59.34%.

### Oil extraction

Oil extraction was carried out using Soxhlet extractor and n-hexane as solvent. Mass of dried spent coffee ground used on oil extraction was 100 g and 750 mL of solvent was used. This process was repeated for 19 times and averaged, oil extraction process takes 89.79 minutes to complete with oil yield of 18.14% by mass of dried spent coffee grounds and 74.23% of solvent recovery.

### Fatty acid profile

**Fig. 2:**
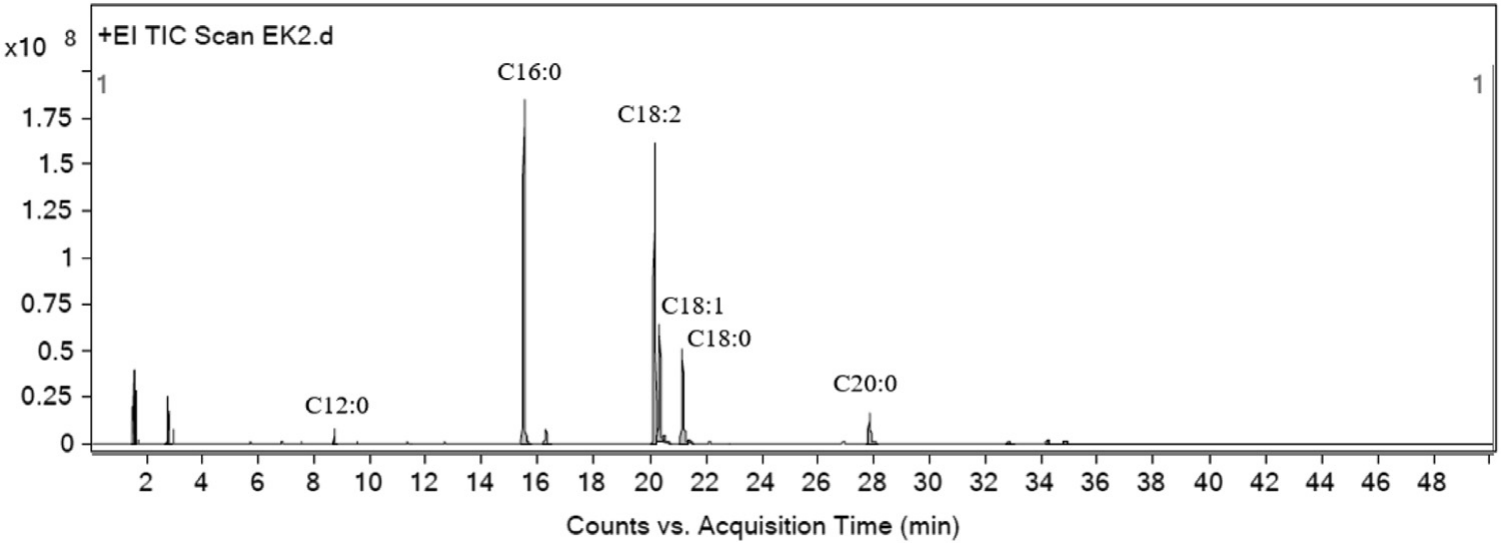
Chromatogram for biodiesel from coffee oil.

After going through the esterification process, the composition of spent coffee grounds oil was measured using a GC-MS. Fig. 2 shows the fatty acids profile. The majority or the fatty acids contained in the oil was palmitic acid and linoleic acid with 33.82% and 32.98% respectively. Followed with 11.76% of oleic acid, 11.24% of stearic acid, 3.94% of arachidic acid, and 0.53% of lauric acid. Spent coffee grounds oil produced contain 49.53% of saturated fatty acid, 44.74% of unsaturated fatty acid, and 5.73% others.

### Properties of biodiesel

The properties of biodiesel produced from this research can be seen in the Table 4 along with the test methods and limitations according to Indonesian biodiesel standards (SNI 7182:2015). There are several properties of biodiesel that do not meet the Indonesian biodiesel standard (SNI 7182: 2015), such as kinematic viscosity, acid number, and oxidation stability. The viscosity of biodiesel was strongly influenced by the methyl ester content, the higher the levels of palmitic acid and stearic acid will increase the viscosity of the oil [38,39]. The acid level in this study can still be lowered again by doing the esterification process for the second time so that the acid level of the oil before entering the transesterification process was already low. The low oxidation stability of coffee grounds biodiesel was because SCG have gone through various heating processes in the presence of oxygen, such as the roasting process which is generally carried out at a temperature of 180 - 240°C, the process of brewing coffee with hot water at high pressure (espresso), the drying process at temperature 105°C, oil extraction process at a temperature of 70 - 75°C. This will greatly affect the oxidation stability of the oil in the coffee grounds. The conversion ratio from coffee oil to biodiesel was 57.32%, namely 45.39 grams of biodiesel produced from 79.18 grams of coffee oil.

**Table 4:** Properties of biodiesel.

Properties	Unit	Method	Limit (SNI 7182:2015)	Value
Density at 15°C	g/mL	ASTM D 1298	0.85 – 0.89	0.887
Kinematic viscosity at 40°C	mm^2^/s	ASTM D 445	2.3 – 6.0	7.67
Acid value	mg KOH/g oil	AOCS Cd 3d-63	Max 0.5	1.19
Saponification value	mg KOH/g oil	AOCS Ca 14-56	-	197.17
Iodine value	%-w (g-I_2_/100g)	AOCS Cd 1-25	Max 115	26.89
Oxidation stability	hours	EN 14112	Min 6	0.1
Higher heating value	MJ/kg	ASTM D 240 – 02 (2007)	-	36.69

### Carbonization and activation process

Both SCG with oil extraction and without oil extraction was carbonized at 500, 600, 700, and 800°C for 30 minutes. Sample A_500 means SCG with oil extraction carbonized at 500°C, and B_600 means SGC without oil extraction carbonized at 600°C. Carbon produced from carbonization process both from A and B type SCG was activated with chemical activation using KOH and heated at 750°C for 2 hours. Table 5 shows the yield from carbonization and activation process.

**Table 5:** Carbonization and activation results.

Sample	Carbonization			Activation			Total yield (% m/m)
	Temperature (˚C)	Duration (minutes)	Yield (% m/m)	Temperature (˚C)	Duration (minutes)	Yield (% m/m)	
A	500	30	32.80	750	120	65.22	21.39
	600	30	30.65			70.89	21.73
	700	30	29.59			72.50	21.45
	800	30	29.13			82.28	23.97
B	500	30	29.44			68.28	20.10
	600	30	27.15			72.06	19.56
	700	30	26.01			76.22	19.82
	800	30	25.69			82.56	21.21

### Properties of activated carbon

The properties of activated carbon produced from this research can be seen in Table 6 along with the test methods and limitation according to Indonesian activated carbon standard, namely SNI 06-3730-1995. The properties of activated carbon measured was moisture content, ash content, volatile matter content, carbon content, iodine number, and methylene blue number. The variation of carbonization temperature and the type of sample used did not really affect the moisture content of activated carbon. The ash content of activated carbon made from sample A was lower when compared to sample B. In addition, the higher carbonization temperature, the higher ash content, this was consistent with the statement of previous study [37]. The volatile matter content will increase by increasing carbonization temperature. The volatile matter content of sample B was higher than sample A. Carbon content will decreased by increasing the carbonization temperature both for sample A and sample B. In Table 6, it can be seen that sample A has a better iodine number than sample B for the same carbonization temperature. The best iodine adsorption was obtained in sample A_700 with a value of 1,224.6 mg/g, and for sample B the best iodine adsorption was obtained in sample B_800 with a value of 966.0 mg/g. Similar results with the adsorption of iodine were also shown for the adsorption of activated carbon against methylene blue. Sample A has a better adsorption ability of methylene blue than sample B. The best adsorption of methylene blue was obtained by sample A_600 with a value of 157.7 mg/g and for sample B the best absorption of methylene blue was obtained by sample B_700 with a value of 141.4 mg/g. Table 6 also shows the yield of activated carbon produced from dried spent coffee grounds. Table 7 shows the comparison of activated produced from this study against other researchers.

**Table 6:** Characteristic of activated carbon.

	Method	SNI 06 – 3730 – 1995	A_500	A_600	A_700	A_800	B_500	B_600	B_700	B_800
Moisture content, %	ASTM D 2867 – 04	Max 15%	2.42%	2.76%	2.06%	1.66%	2.50%	2.08%	2.24%	1.79%
Ash content, %	ASTM D 2866 – 94	Max 10%	0.34%	3.36%	3.01%	5.22%	0.57%	4.31%	4.48%	5.67%
Volatile matter content, %	ASTM D 5832 – 98	Max 25%	13.84%	17.12%	18.19%	21.89%	15.84%	18.77%	20.41%	23.68%
Carbon content, %	-	Min 65%	83.40%	76.76%	76.74%	71.23%	81.09%	74.84%	72.87%	68.86%
Iodine number, mg/g	ASTM D 4607 – 94	Min 750 mg/g	801.2	938.0	1,224.6	1,013.8	725.8	804.8	934.9	966.0
Methylen blue number, mg/g	-	Min 120 mg/g	96.2	157.7	153.1	152.0	61.7	129.9	141.4	133.3
Yield, %	-	-	21.39%	21.73%	21.45%	23.97%	20.10%	19.56%	19.82%	21.21%

**Table 7:** Comparison of the resulting activated carbon against other researchers.

Study	Raw material	Moisture content (%)	Ash content (%)	Volatile matter content (%)	Carbon content (%)	Iodine number (mg/g)	Methylene blue number (mg/g)
Experiment	Spent coffee grounds (arabica)	1.66 – 2.76	0.34 – 5.67	13.84 – 23.68	68.86 – 83.40	725.8 – 1,224.6	61.7 – 157.7
Yuliusman et al. [40]	Spent coffee grounds (robusta)	-	-	-	-	196.61 – 432.60	-
Mariana et al. [41]	Spent coffee grounds	6.38	1.05	-	-	856.58	-
Ren et al. [42]	Spent coffee grounds	-	-	-	-	1,398.4	270.32
Rhaman et al. [43]	Rice husk	-	-	-	-	395 ± 5	48 ± 1
Shrestha et al. [44]	Rice husk	-	-	-	-	863 - 1,726	80 - 608
Yusufu et al. [45]	Coconut shell Wood	6.4 9.4	2.8 1.8	-	-	848.69 914.17	-
Huang et al. [46]	Coconut shell	-	2.96 – 3.32	-	-	340 – 780	-
Andas et al. [47]	Palm shell	-	-	-	-	994.83	-
Kouotou et al. [48]	Palm shell	-	-	-	-	697.86	346.25
Santana et al. [49]	Bamboo	-	-	-	-	-	298.82
Wu et al. [50]	Bamboo	-	-	-	-	915	269
Lori et al. [51]	Bagasse Sorghum Straw	-	-	-	-	626 667 593	502 662 390

**Fig. 3:**
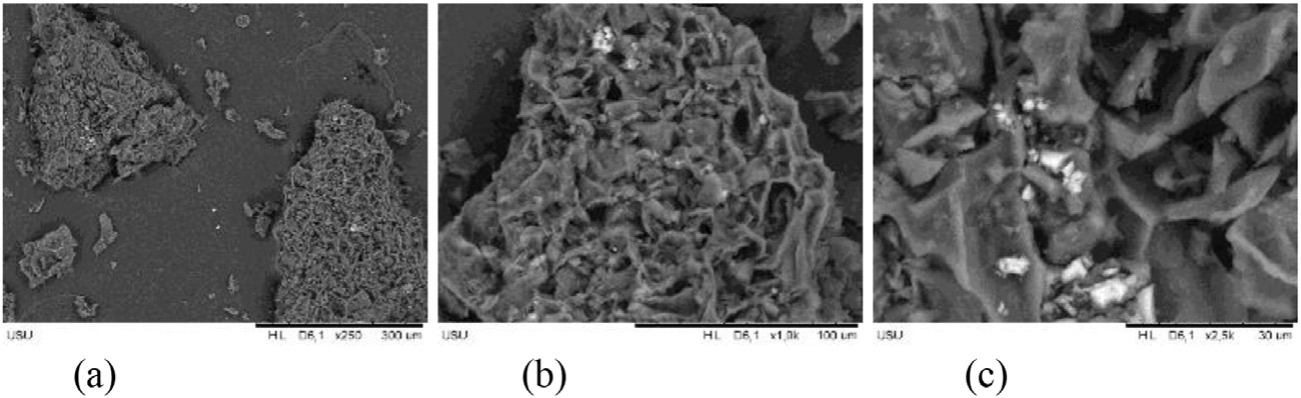
SEM characterization for sample A_700 (a) 250x magnification, (b) 1000x magnification, (c) 2500x magnification.

**Fig. 4:**
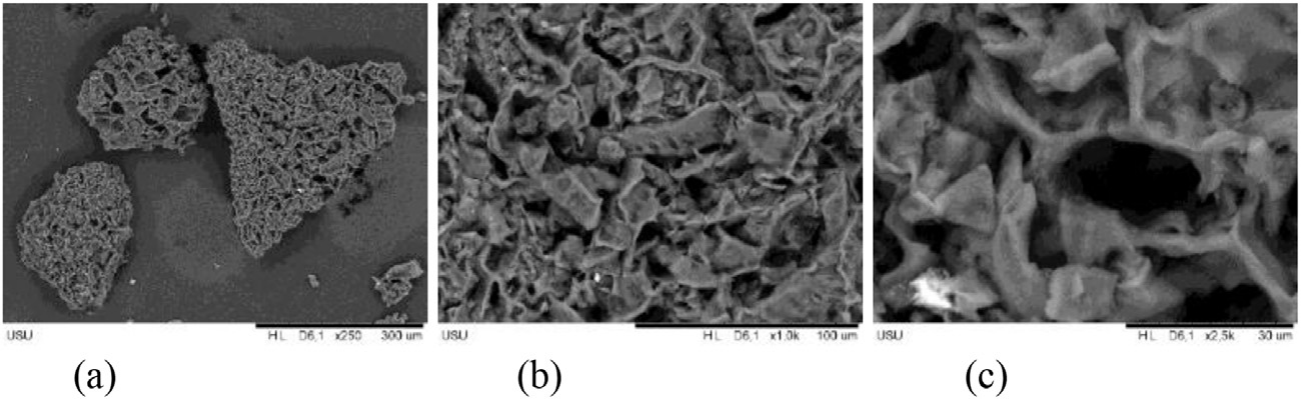
SEM characterization for sample B_800 (a) 250x magnification, (b) 1000x magnification, (c) 2500x magnification.

The pore structure of the resulting activated carbon can be seen using SEM (Scanning Electron Microscopy). Fig. 3 shows the SEM characterization for sample A_700 and Fig. 4 shows the SEM characterization for sample B_800. From the SEM results, it can be seen that sample A_700 has a tighter pore structure so that it has a larger surface area when compared to sample B_800. The larger the surface area of activated carbon, the contact area between the activated carbon and the substance to be adsorbed will also increase, this will lead to better adsorption ability of iodine and methylene blue.

## Conclusion

Biodiesel produced from spent coffee grounds still does not fully meet the Indonesia biodiesel standards (SNI 7182:2015) because the values of kinematic viscosity, acid content, and oxidation stability are still outside the standard limits. The process of extracting oil from coffee grounds affects the quality and quantity of activated carbon produced where by extracting the oil will increase the adsorption of activated carbon against iodine and methylene blue, reduce ash and volatile matter content thus increase carbon content from activated carbon. By increasing the carbonization temperature from 500°C to 800°C causes an increase in the ash and volatile matter content, and a decrease in carbon content. The moisture content was not significantly affected by the increase in carbonization temperature. Activated carbon that meets the quality requirements of Indonesian activated carbon (SNI 06 - 3730 - 1995) was activated carbon made from coffee grounds by oil-extracted and non oil-extracted at carbonization temperatures of 600, 700, and 800°C. The best quality of activated carbon from adsorption ability was obtained from coffee grounds that have been oil-extracted and the carbonization process at 700°C for iodine absorption of 1224.59 mg/g, and carbonization at 600°C for methylene blue adsorption of 157.68 mg/g.

## CRediT author statement

Jefry Kusuma: Conceptualization, experiment, testing, analysing, writing

Yuli S. Indartono: Supervision, guidance, writing-reviewing

Didin Mujahidin: Supervision, guidance, writing-reviewing

## Declaration of Competing Interest

The authors declare that they have no known competing financial interests or personal relationships that could have appeared to influence the work reported in this paper.

## Data Availability

Data will be made available on request.
